# Revisiting annual screening for latent tuberculosis infection in healthcare workers: a cost-effectiveness analysis

**DOI:** 10.1186/s12916-017-0865-x

**Published:** 2017-05-17

**Authors:** Guillaume A. Mullie, Kevin Schwartzman, Alice Zwerling, Dieynaba S. N’Diaye

**Affiliations:** 1 Respiratory Epidemiology and Clinical Research Unit, Montreal Chest Institute, Montreal, QC Canada; 2 Faculty of Medicine, McGill University, Montreal, QC Canada; 3 McGill International TB Centre, McGill University, Montreal, Quebec Canada; 4 McGill University Health Centre, 1001 boulevard Décarie, Room D05.2511, Montreal, H4A 3J1 Quebec Canada; 5 Bloomberg School of Public Health, Johns Hopkins University, Baltimore, Maryland USA

**Keywords:** Latent tuberculosis, Health personnel, Screening, Cost-effectiveness

## Abstract

**Background:**

In North America, tuberculosis incidence is now very low and risk to healthcare workers has fallen. Indeed, recent cohort data question routine annual tuberculosis screening in this context. We compared the cost-effectiveness of three potential strategies for ongoing screening of North American healthcare workers at risk of exposure. The analysis did not evaluate the cost-effectiveness of screening at hiring, and considered only workers with negative baseline tests.

**Methods:**

A decision analysis model simulated a hypothetical cohort of 1000 workers following negative baseline tests, considering duties, tuberculosis exposure, testing and treatment. Two tests were modelled, the tuberculin skin test (TST) and QuantiFERON®-TB-Gold In-Tube (QFT). Three screening strategies were compared: (1) annual screening, where workers were tested yearly; (2) targeted screening, where workers with high-risk duties (e.g. respiratory therapy) were tested yearly and other workers only after recognised exposure; and (3) post exposure-only screening, where all workers were tested only after recognised exposure. Workers with high-risk duties had 1% annual risk of infection, while workers with standard patient care duties had 0.3%. In an alternate higher-risk scenario, the corresponding annual risks of infection were 3% and 1%, respectively. We projected costs, morbidity, quality-adjusted survival and mortality over 20 years after hiring. The analysis used the healthcare system perspective and a 3% annual discount rate.

**Results:**

Over 20 years, annual screening with TST yielded an expected 2.68 active tuberculosis cases/1000 workers, versus 2.83 for targeted screening and 3.03 for post-exposure screening only. In all cases, annual screening was associated with poorer quality-adjusted survival, i.e. lost quality-adjusted life years, compared to targeted or post-exposure screening only. The annual TST screening strategy yielded an incremental cost estimate of $1,717,539 per additional case prevented versus targeted TST screening, which in turn cost an incremental $426,678 per additional case prevented versus post-exposure TST screening only. With the alternate “higher-risk” scenario, the annual TST strategy cost an estimated $426,678 per additional case prevented versus the targeted TST strategy, which cost an estimated $52,552 per additional case prevented versus post-exposure TST screening only. In all cases, QFT was more expensive than TST, with no or limited added benefit. Sensitivity analysis suggested that, even with limited exposure recognition, annual screening was poorly cost-effective.

**Conclusions:**

For most North American healthcare workers, annual tuberculosis screening appears poorly cost-effective. Reconsideration of screening practices is warranted.

## Background

Tuberculosis (TB) infection has long been considered a hazard for healthcare workers (HCWs), where occupational factors such as caring for patients with respiratory TB increase risk [1–3]. Routine screening and treatment of latent TB infection (LTBI) has traditionally been an important element of TB prevention measures in Canadian and US healthcare institutions.

The preferred method of serial screening for LTBI in Canadian HCWs is the tuberculin skin test (TST), with the recommended frequency of testing reflecting the volume of TB patients cared for at the healthcare facility and the risk inherent to specific work activities. Baseline two-step tuberculin testing is recommended for all HCWs upon hiring in Canada [4]. Subsequent annual testing is recommended for those with intermediate-risk duties (e.g. direct patient care) in settings where TB patients are more likely to be encountered. Annual testing is also recommended for all HCWs who perform high-risk duties (e.g. cough-inducing procedures or laboratory procedures with potential *M. tuberculosis* exposure), regardless of work setting. Other HCWs, who are at lower risk based on setting and/or duties, are retested only after identified exposure where there is concern about possible transmission [4]. US guidelines are similar, although they suggest that an interferon-gamma release assay (IGRA) may replace the TST [5].

Recent data call into question the use of routine serial testing for the ‘typical’ North American HCW involved in most patient care activities which may entail exposure to *M. tuberculosis*. This is because false-positive test conversions become more frequent than true-positives as the risk of true infection falls, regardless of the specific test used. The true risk now faced by most North American HCWs is small, as TB incidence in the US is now at an all-time low. This is concordant with reported estimated conversion rates of 0.8–0.9% over 6–18 months of follow-up [6, 7]. Several reports have also suggested more frequent false-positive ‘conversions’ with the IGRAs [6, 8–11], although one study reported lower apparent conversion rates with the T-SPOT.TB test, particularly when borderline results were excluded [7].

Surveillance data in the US (1995–2007) suggest that overall rates of active TB among HCWs have fallen to that of the general population, with the major risk factor being foreign birth [12]. The large and frequent nosocomial epidemics of the 1980s and 1990s no longer occur. Most TB now arising among North American HCWs likely reflects non-occupational exposure, most often in other countries before immigration. This reinforces the importance of baseline worker screening, but casts further doubt on the need for routine serial testing.

Using a decision analysis model, we compared the cost-effectiveness of current serial screening practices for LTBI among North American HCWs who work in settings where they may encounter TB patients, with that of more targeted approaches, where only HCWs involved in high-risk activities undergo routine annual testing, or where all workers are retested only after identified TB exposures. We did not evaluate the yield or cost-effectiveness of worker screening at hiring – this remains important to detect previously acquired latent infection, notably among foreign-born workers and also as a baseline in the event that repeat testing is considered after identified exposure.

## Methods

### Model overview

**Fig. 1:**
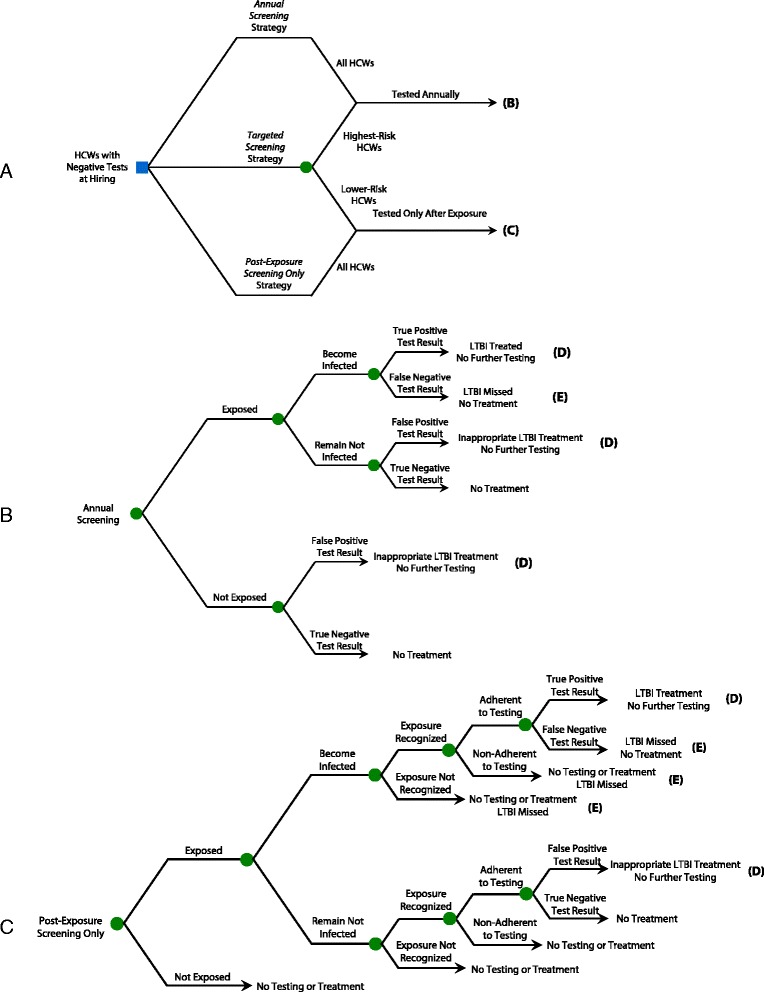
Decision tree model: overview of main screening strategies. *HCWs* healthcare workers, *TB* tuberculosis, *LTBI* latent TB infection, *INH* isoniazid. **Path A** Schematic overview of the basic strategies. For simplicity of display, the targeted screening strategy is divided into two branches. The upper branch of that strategy indicates that the highest-risk workers undergo annual screening; the lower branch indicates that lower-risk workers undergo post-exposure screening only. The two other main strategies (annual screening and post-exposure screening only) both apply a single screening protocol to all workers, regardless of their risk profile. **Path B** Subtree for annual screening. Workers with negative tests remain eligible for screening the following year. **Path C** Subtree for post-exposure screening. With this strategy, the steps that lead to testing for infection after exposure include the probability that exposure occurs, the probability that it leads to infection, the probability that it is recognised, and the probability that a worker presents for testing after being instructed to do so in the context of a recognised exposure. In the event that an exposure occurs but is not recognised, no testing takes place, and new infection is missed. Workers who are not tested, or who have negative tests, remain eligible for screening in case of subsequent exposure

We constructed a decision analysis model using TreeAge software (TreeAge Pro 2015, Health Care Edition, Williamstown, MA). It followed a hypothetical cohort of 1000 HCWs after negative baseline tests at hiring, including workers with intermediate- and high-risk duties, to estimate costs, clinical outcomes, and quality-adjusted survival (QALYs) associated with broad-based versus targeted annual screening. The sequence of events, from hiring onward, was modelled with respect to TB exposure, testing and treatment. Figure 1 shows a simplified schematic of the decision tree.

**Fig. 2:**
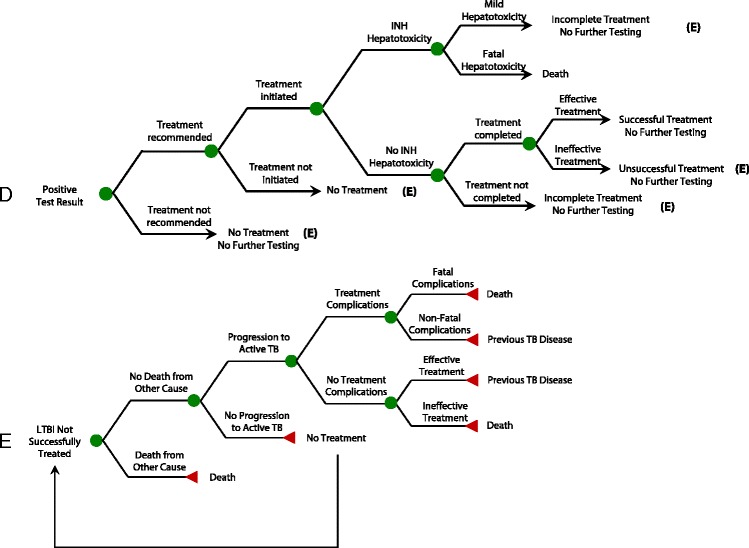
Decision tree model: simplified treatment subtrees. *HCWs* healthcare workers, *TB* tuberculosis, *LTBI* latent TB infection, *INH* isoniazid. **Path D** Simplified treatment subtree, for patients who are treated for LTBI after test conversion. For HCWs who are inappropriately treated for LTBI after false-positive test conversions, the subtree is identical to the one displayed in Path D, with the exception that there is no benefit from treatment since workers do not truly have LTBI. These HCWs are no longer eligible for subsequent screens, leading to missed opportunities for prevention if true infection occurs in the future. **Path E** Subtree for LTBI not successfully treated. This relates to workers who receive no treatment, incomplete treatment, or unsuccessful treatment for latent TB infection. It is assumed that all active TB cases are diagnosed and fully treated

Two screening tests were considered: the TST and the QuantiFERON®-TB-Gold (QFT). The cohort was assumed to have a mean age of 35 and to be 80% female, consistent with previous reports [13–15]. All HCWs were assumed to have previously tested negative in a baseline screen performed at hiring. HCWs were subsequently retested either yearly or only after a recognised TB exposure (Figs. 1 and 2). We considered two scenarios for the risk of TB exposure. The primary (base case) scenario reflected more recent reports from the US, while the alternate scenario involved higher risk estimates reflecting observations in the US during the 1980s and 1990s.

### Strategies

#### Annual screening – TST or QFT

Current practices for HCWs with a negative baseline test involve an annual test for HCWs involved in intermediate-risk duties (in settings where TB patients may be encountered) and for HCWs performing high-risk duties (in all healthcare settings). HCWs who convert their test from negative to positive are offered isoniazid for LTBI once active TB is excluded.

#### Targeted screening – TST or QFT

Only HCWs with high-risk duties (in all healthcare settings) are screened annually after negative baseline tests. All others are retested only if an unprotected exposure to a contagious TB patient is recognised. HCWs who convert their test from negative to positive are offered isoniazid for LTBI once active TB is excluded.

#### Post-exposure screening only – TST or QFT

To assess the impact of screening high-risk HCWs annually, we also evaluated a strategy where all HCWs with negative baseline tests are retested only after recognised exposure to contagious TB, regardless of duties. HCWs who convert their test from negative to positive are offered isoniazid for LTBI, once active TB is excluded.

### Key variables and model assumptions

In the base case analysis, 27% of HCWs at risk of TB exposure were assumed to perform high-risk duties, i.e. cough-inducing procedures, myobacteriology or pathology laboratory work [16]. They were assumed to have a 4.4% annual probability of exposure to contagious TB, corresponding to the ‘moderate-risk’ group described by Salpeter et al. in 2004 [17], and reflecting more recent incidence data reported by Lambert et al. in 2012 [12]. In the alternate scenario, they were assumed to have a 13.1% annual probability of exposure, corresponding to the ‘high-risk’ group described by Salpeter et al. [17, 18].

All other workers were assumed to perform intermediate-risk tasks, i.e. general patient care, with a 1.3% annual risk of significant exposure, corresponding to Salpeter’s ‘low-risk’ group [12, 17]; in the alternate scenario, the risk of exposure for intermediate-risk workers was increased to 4.4%, corresponding to Salpeter’s ‘moderate-risk’ group [17]. After exposure to a contagious TB patient, workers had a 22.9% probability of becoming infected [18]. For the base case scenario, this yielded estimated annual risks of infection of 1% for the highest risk workers and 0.3% for the intermediate-risk workers. In the alternate scenario, the corresponding annual risks of infection were 3% and 1%, respectively.

Since our focus was HCWs at risk of developing TB infection, we did not consider the lowest-risk workers, clerical, maintenance and laundry employees [16]. Similarly, we assumed that all HCWs in the cohort worked in hospitals where TB patients were likely to be admitted (i.e. settings not already considered low risk), and they would be screened annually based on current practices.

In the base-case analysis, we assumed that 75% of exposures conferring any relevant risk of transmission were identified and acted upon accordingly. The probability of testing, either annually or after exposure, reflected workers’ adherence to screening protocols. With the annual screening strategy, we assumed that annual testing was mandatory for renewal of employment; therefore, workers were 100% adherent – a conservative assumption that would tend to overestimate benefits of annual screening. For testing of workers following identified exposures, we assumed that 88.8% adhered to screening with the TST, as in the study by LoBue et al. [19], and this increased to 95% for the QFT, reflecting better reported adherence with the QFT (there is no need to return for reading) [20].

The annual risk of progression to active TB for infected workers was 2.5% if infection was recent (within the last 2 years) and 0.1% if more longstanding [21, 22]. Where applicable, test characteristics for TST and QFT were taken from a systematic review of the performance of these tests [23] and from studies reporting results from serial testing [6, 11]. Key parameter values are presented in Table 1, including cost variables related to screening, diagnostics, treatment and follow-up for latent and active TB. The cost values reflect published North American data.

**Table 1:** Model parameters: base-case values, assumptions and ranges used in the sensitivity analyses

Parameter	Base-case value	Range	References
Prevalence of latent tuberculosis infection (LTBI) at the time of hire (before baseline testing)	10.0%	(10–30%)	[6]
Probability of being recently infected among those with LTBI at baseline	16.7%	(10–30%)	Model assumption
Proportion of healthcare workers (HCWs) performing high-risk activities	27.0%	(10–30%)	[16]
Annual risk of TB exposure in HCWs performing high-risk activities:			
Base-case scenario	4.4%	(0–40%)	[12, 17]
Alternate scenario assuming higher risks	13.1%	(0–40%)	[17, 18]
Annual risk of TB exposure in HCWs performing intermediate-risk activities:			
Base-case scenario	1.3%	(0–15%)	[12, 17]
Alternate scenario assuming higher risks	4.4%	(0–15%)	[17, 18]
Probability of acquiring new TB infection given exposure	22.9%	(0–30%)	[18]
Adherence of HCWs to annual screening (mandatory for continued employment)	100%	(50–100%)	Model assumption
Probability that TB exposure is recognised	75.0%	(50–100%)	Model assumption
Probability of being screened after TB exposure is recognised (tuberculin skin test)	88.8%	(50–100%)	[19]
Probability of being screened after TB exposure is recognised (QuantiFERON®-TB-Gold)	95.0%	(50–100%)	[20]
Sensitivity of tuberculin skin test in serial testing	95.0%	(70–100%)	[26, 29]
Sensitivity of QuantiFERON®-TB-Gold in serial testing	95.0%	(70–100%)	[27, 28]
Specificity of tuberculin skin test for serial testing, after baseline negative test	97.0%	(70–100%)	[23]
Specificity of QuantiFERON®-TB-Gold for serial testing after baseline negative test	95.0%	(70–100%)	[6, 11]
Efficacy of isoniazid preventive treatment	90.0%	(80–100%)	[42]
Probability that isoniazid is recommended to worker after conversion on repeat testing	100%	(50–100%)	Model assumption
Probability that worker with conversion starts isoniazid treatment, after recommendation to take it	82.9%	(50–100%)	[43]
Probability that isoniazid treatment is completed, once started	47.3%	(40–100%)	[43]
Risk of mild isoniazid-induced hepatitis	0.1%		[44]
Risk of fatal isoniazid-induced hepatitis	0.002%		[44]
Annual risk of progression from LTBI to active TB for recently infected (≤2 years since onset of infection)	2.5%	(0–2.5%)	[21]
Annual risk of progression from LTBI to active TB for remotely infected (>2 years since onset of infection)	0.1%		[22]
Risk of death from active TB	4.6%	(0–10%)	[45]
Risk of major adverse event with treatment for active TB	5.1%		[44]
Risk of death, given major adverse event with treatment for active TB	1.5%		[44]
Costs (in 2015 CAN dollars; $1 CAN = $0.77 US)			
Diagnosis for active TB disease	$354		[46–48]
Inpatient treatment of active TB disease	$13,063		[49]
Outpatient treatment of active TB disease	$3,748		[50]
Tuberculin skin test	$15	($10–30)	[51]
QuantiFERON®-TB-Gold	$50	($10–50)	[51]
Complete treatment for LTBI	$591		[52]
Incomplete treatment for LTBI	$272		[52]
Isoniazid-induced hepatitis (mild)	$400		[53]
Isoniazid-induced hepatitis (fatal)	$13,078		[53]
Quality of life adjustments: QALYs lost per year			
Active TB disease treatment	0.15	(0.10–0.30)	[24, 25]
Latent TB treatment	0.03	(0–0.05)	[24, 25]

For calculations of quality-adjusted life years (QALYs), active TB treatment was attributed 0.15 QALYs lost over 1 year. This reflected our published data using the standard gamble, among persons treated for active TB in Montreal [24]. Similarly, based on our previous direct measurements, we attributed a QALY decrement of 0.03 to treatment of LTBI [24]; this was in fact identical to the value used by Shepardson et al. [25]. We did not attribute separate quality weights to adverse drug effects or hospitalisation, since patients both with and without these were included in our previous sample [24]. No quality decrement was associated with untreated LTBI.

For the TST, we assumed that a two-step test was performed at hiring to account for boosting from previous BCG vaccination [26], so only individuals with negative baseline two-step tests (i.e. no boosting) were eligible for subsequent testing. The specificity attributed to serial TSTs was therefore taken from studies performed on non-BCG vaccinated individuals [23]. Although 10% of HCWs were assumed to have LTBI at the time of hiring [2], only 5% of infected workers had false negative baseline tests [27–29] so that only 0.6% of workers with negative baseline tests had undetected LTBI at the beginning of the simulation.

We assumed that all active TB arising among HCWs was ultimately diagnosed and fully treated.

### Outcomes and sensitivity analyses

For each strategy, the model tabulated the expected TB-related costs (in 2015 Canadian dollars; $1 Canadian = $0.77 US), QALYs, the number of new active TB cases, TB-related mortality, and measures of test performance (number of false and true positives) for the HCW cohort, beginning after negative baseline tests at hiring and continuing for the subsequent 20 years (a time horizon that reflects the longest follow-up in clinical trials of isoniazid for LTBI [30]. We estimated incremental cost-effectiveness ratios (ICERs), defined as the additional cost per QALY gained or per additional TB case prevented compared to the next most expensive strategy. The analysis was conducted from the healthcare system perspective, using a 3% annual discount rate for costs and clinical outcomes [31].

To assess the impact of input parameter uncertainties, extensive one-way sensitivity analyses were performed. We varied parameter values across plausible ranges (Table 1). For the most influential variables, we identified thresholds at which the base-case results would change. We also explored two alternate scenarios in further detail, namely (1) we estimated QALYs, and incremental cost per QALY gained, with the assumption that there was no loss of QALYs with uncomplicated treatment of LTBI; and (2) we estimated costs and expected outcomes when a second positive QFT was required to confirm a newly positive QFT on routine serial testing, before proceeding to treatment of LTBI. With this scenario, a confirmatory test was not required when testing was triggered by suspected exposure. This scenario therefore involved an increase in specificity, reduction in sensitivity, plus changes in cost (more for screening, less for downstream investigation and treatment) for routine QFT screens.

To examine the relative influence of uncertainty in the various model inputs, we performed tornado analyses using QALYs and TB cases as outcomes. We also conducted two-way sensitivity analyses combining key parameters.

## Results

### Base-case analysis

For workers with negative baseline tests, the annual screening strategy was estimated to prevent less than one active TB case per 5000 workers screened over 20 years, compared to the targeted strategy where only workers at the highest risk undergo annual screening (Table 2). Moreover, annual screening was associated with a small decrease in quality-adjusted survival compared to targeted screening. The impact of the targeted strategy was similarly limited, when compared to testing all workers only after recognised exposures. Targeted screening was likewise associated with a decrease in quality-adjusted survival compared to post-exposure screening only (Tables 2 and 3). In each case, screening using QFT was associated with slightly poorer quality-adjusted survival than with the TST because of the larger number of false-positive tests leading to treatment for LTBI (Tables 2 and 3).

**Table 2:** Projected outcomes of six TB screening strategies for a cohort of 1000 healthcare workers over 20 years: base-case scenario

Strategy	Cost in 2015 $CAN	QALYs	New active TB cases	Deaths due to active TB	Deaths due to adverse event to treatment of active TB	Deaths due to adverse event to treatment of LTBI	True positive results	False positive results
Post-exposure screening only								
Tuberculin Skin Test	$66,387	15,239.98	3.03	0.13	0.0023	0.00036	63	6
QuantiFERON®-TB-Gold	$77,521	15,239.85	2.97	0.13	0.0023	0.00040	67	11
Targeted screening								
Tuberculin Skin Test	$151,517	15,237.96	2.83	0.12	0.0022	0.00093	67	109
QuantiFERON®-TB-Gold	$263,660	15,236.90	2.86	0.13	0.0022	0.00120	63	161
Annual screening								
Tuberculin Skin Test	$404,956	15,231.85	2.68	0.12	0.0020	0.00258	75	413
QuantiFERON®-TB-Gold	$817,695	15,227.92	2.80	0.12	0.0021	0.00362	64	607

*TB* tuberculosis, *QALYs* quality-adjusted life years, *LTBI* latent tuberculosis infection

**Table 3:** Projected outcomes of six TB screening strategies for a cohort of 1000 healthcare workers over 20 years: alternate scenario with increased worker risk

Strategy	Cost in 2015 $CAN	QALYs	New active TB cases	Deaths due to active TB	Deaths due to adverse event to treatment of active TB	Deaths due to adverse event to treatment of LTBI	True positive results	False positive results
Post-exposure screening only								
Tuberculin Skin Test	$198,480	15,234.05	8.90	0.39	0.0068	0.00109	195	17
QuantiFERON®-TB-Gold	$228,809	15,233.75	8.73	0.38	0.0067	0.00119	201	30
Targeted screening								
Tuberculin Skin Test	$257,670	15,232.84	8.18	0.36	0.0063	0.00152	193	96
QuantiFERON®-TB-Gold	$365,397	15,231.90	8.23	0.36	0.0063	0.00177	184	146
Annual screening								
Tuberculin Skin Test	$487,837	15,227.38	7.64	0.33	0.0058	0.00307	203	373
QuantiFERON®-TB-Gold	$868,662	15,223.94	7.95	0.35	0.0061	0.00395	174	553

*TB* tuberculosis, *QALYs* quality-adjusted life years, *LTBI* latent tuberculosis infection

The least costly screening strategy was post-exposure screening only with the TST, and the most costly was annual screening with the QFT. For the TST, the estimated incremental cost of the annual screening strategy was $1,717,539 per additional active TB case prevented, relative to the targeted strategy (Table 4). The corresponding ICER for targeted screening with TST compared to post-exposure screening only (with TST) was $426,678 per additional case prevented. Of note, with post-exposure screening only, the QFT prevented slightly more cases than the TST, at a cost of $197,017 per additional case prevented. For the targeted and annual screening strategies, the QFT was dominated, meaning that it prevented fewer cases than the TST, but at higher cost.

**Table 4:** Cost-effectiveness of six TB screening strategies for a cohort of 1000 healthcare workers over 20 years: base-case scenario

Strategy	Cost in 2015 $Can	Incremental cost	QALYs	Increment in QALYs	Incremental cost per QALY gained^a^	New active TB cases	Increment in active TB cases prevented	Incremental cost per additional TB case prevented^a^
Post-exposure screening only								
Tuberculin Skin Test	$66,387	—	15,239.98	––	––	3.03	—	—
QuantiFERON®-TB-Gold	$77,521	$11,134	15,239.85	–0.13	–– (Dominated^b^)	2.97	0.06	$197,017
Targeted screening								
Tuberculin Skin Test	$151,517	$85,130	15,237.96	–2.02	–– (Dominated^b^)	2.83	0.14	$517,437^c^
QuantiFERON®-TB-Gold	$263,660	$197,273	15,236.90	–3.07	–– (Dominated^b^)	2.86	–0.04	— (Dominated^b^)
Annual screening								
Tuberculin Skin Test	$404,956	$338,569	15,231.85	–8.12	–– (Dominated^b^)	2.68	0.15	$1,717,539
QuantiFERON®-TB-Gold	$817,695	$751,308	15,227.92	–12.06	–– (Dominated^b^)	2.80	–0.12	— (Dominated^b^)

*TB* tuberculosis, *QALYs* quality-adjusted life years

^a^Relative to next most expensive, non-dominated strategy

^b^Dominated because it is more expensive and less effective than the proposed alternative; hence no incremental cost-effectiveness ratios are provided

^c^Incremental cost of $426,678 per additional TB case prevented, relative to post-exposure TST screening only

The three strategies were nearly identical with respect to expected deaths related to TB or TB treatment, which were extremely uncommon. However, when screening in the absence of recognised exposure, there was a large number of false-positive tests: with both the annual and targeted screening strategies, this number in fact exceeded the expected number of true-positive results (Table 2). Another noteworthy result was that the yield of true positives decreased with more intensive screening with the QFT. This reflected the fact that workers with false-positive tests were no longer eligible for subsequent screening, regardless of later exposures (Table 2).

### Alternate scenario with higher worker risks

With the alternate scenario involving higher risks to workers, the estimated incremental cost of the annual screening strategy with TST was $426,678 per additional active TB case prevented, relative to the targeted strategy (Table 5). The corresponding ICER for targeted screening compared to post-exposure screening only was $52,552 per additional case prevented (Table 5). Strategies using the QFT were consistently more expensive than the corresponding strategies using the TST. Moreover, the QFT did not prevent any additional TB cases, for the annual and targeted screening strategies (Table 5).

**Table 5:** Cost-effectiveness of six TB screening strategies for a cohort of 1000 healthcare workers over 20 years: alternate scenario with increased worker risk

Strategy	Cost in 2015 $Can	Incremental cost	QALYs	Increment in QALYs	Incremental cost per QALY gained^a^	New active TB cases	Increment in active TB cases prevented	Incremental cost per additional TB case prevented^a^
Post-exposure screening only								
Tuberculin Skin Test	$198,480	—	15,234.05	—	—	8.90	—	—
QuantiFERON®-TB-Gold	$228,809	$30,329	15,233.75	–0.30	— (Dominated^b^)	8.73	0.17	— (Extended dominance^c^)
Targeted screening								
Tuberculin Skin Test	$257,670	$59,190	15,232.84	–1.22	— (Dominated^b^)	8.18	0.55	$52,552
QuantiFERON®-TB-Gold	$365,397	$166,917	15 231.90	–2.15	— (Dominated^b^)	8.23	–0.05	— (Dominated^b^)
Annual screening								
Tuberculin Skin Test	$487,837	$289,357	15,227.38	–6.68	— (Dominated^b^)	7.64	0.54	$426,678
QuantiFERON®-TB-Gold	$868,662	$670,182	15,223.94	–10.11	— (Dominated^b^)	7.95	–0.31	— (Dominated^b^)

*TB* tuberculosis, *QALYS* quality-adjusted life years

^a^Relative to next most expensive, non-dominated strategy

^b^Dominated because it is more expensive and less effective than the proposed alternative; hence no ICER is provided

^c^Extended dominance by the targeted tuberculin skin testing screening strategy, meaning that the incremental cost-effectiveness ratio is in fact lower for the targeted tuberculin skin testing screening strategy, so the targeted strategy is preferred. Hence no ICER is provided

### Sensitivity analyses

**Fig. 3:**
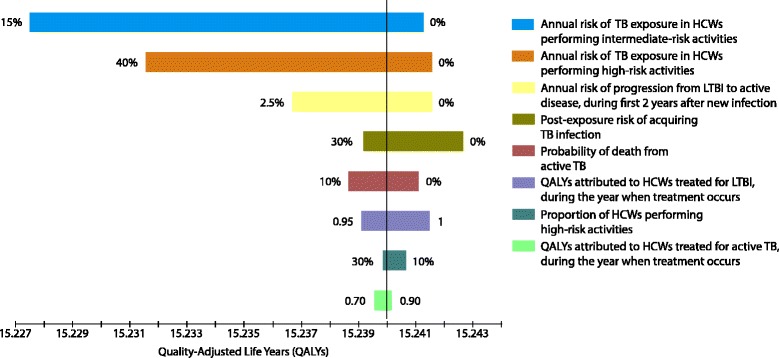
Tornado analysis of expected QALYs per worker over 20 years: 3% discount rate. *TB* tuberculosis, *HCWs* healthcare workers, *LTBI* latent tuberculosis infection, *QALYs* quality-adjusted life years. The most influential input variables were risk of TB exposure (especially for intermediate-risk HCWs), risk of progression to active TB following new infection and risk of infection after exposure, risk of death with active TB, QALY decrements attributed to latent TB treatment and active TB, and proportion of workers performing high-risk duties

**Fig. 4:**
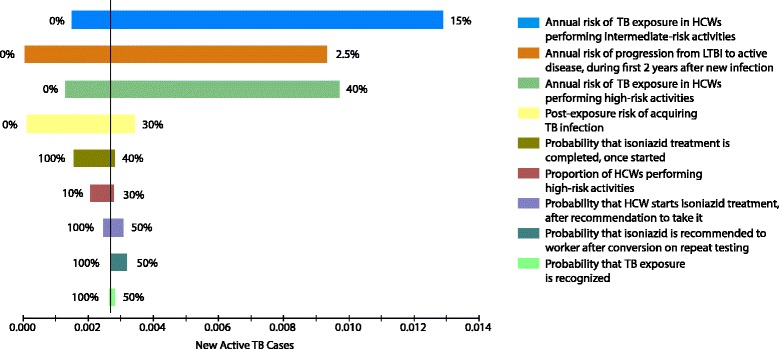
Tornado analysis of expected TB cases per worker over 20 years: ﻿3% discou﻿nt rate. *TB* tuberculosis, *HCWs* healthcare workers, *LTBI* latent tuberculosis infection. The most influential input variables were risk of TB exposure (especially for intermediate-risk HCWs), risk of progression to active TB following new infection and risk of infection after exposure, proportion of workers performing high-risk duties, proportions of workers prescribed, initiating, and completing isoniazid treatment when indicated, and the proportion of exposures which are recognised

With respect to expected QALYs, the most influential input variables were risk of TB exposure (especially for intermediate-risk HCWs), risk of progression to active TB following new infection and risk of infection after exposure, QALY decrements attributed to latent TB treatment and active TB, proportion of workers performing high-risk duties, and risk of death with active TB (Fig. 3). With respect to expected TB cases, the most influential variables were largely similar (except for QALY decrements), with the addition of the proportions of workers prescribed, initiating, and completing isoniazid treatment when indicated, and the proportion of exposures which are recognised (Fig. 4).

When we attributed no QALY decrement to treatment of LTBI, but kept all other parameters from our base case scenario, annual screening with the TST cost an estimated $3,314,149 per QALY gained, compared to targeted screening with the TST. Targeted screening with TST cost an estimated $862,553 per QALY gained, compared to post-exposure screening with TST only. Post-exposure screening with the QFT cost an estimated $372,961 per QALY gained, compared to post-exposure screening with the TST. Targeted and annual screening with QFT were more expensive and associated with fewer expected QALYs, i.e. they were dominated strategies (detailed data not shown).

**Fig. 5:**
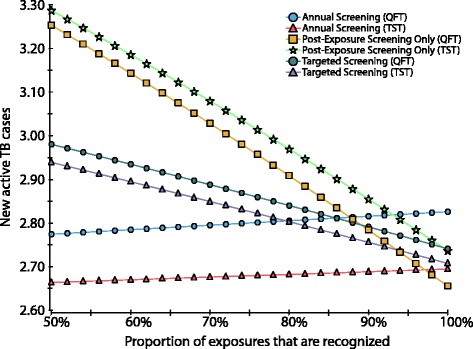
One-way sensitivity analysis evaluating the impact of recognition of exposures on effectiveness (new active TB cases over 20 years). *TB* tuberculosis, *TST* Tuberculin Skin Test, *QFT* QuantiFERON®-TB-Gold. The proportion of exposures that are recognised strongly influences the relative effectiveness of the screening strategies. When most exposures are missed, the annual screening strategy is much more effective than both targeted screening and post-exposure screening only. However, as the proportion of recognised exposures increases, the targeted strategies become progressively more effective at preventing new active TB cases. Even if only 50% of exposures are recognised, annual screening with TST costs an estimated $927,242 per additional TB case prevented, relative to the targeted TST screening strategy

The proportion of exposures which are recognised affected the ranking of the screening strategies with respect to TB cases prevented. When most exposures are missed, the annual screening strategy prevents more TB cases than both targeted screening and post-exposure screening only (Fig. 5). However, even if only 50% of exposures are recognised, annual screening with TST costs an estimated $927,242 per additional TB case prevented, relative to the targeted TST screening strategy. If a lower proportion of workers perform high-risk duties, annual screening becomes even less cost-effective. For example, if only 5% of workers perform high-risk duties, annual screening with TST costs an estimated $1,779,560 per additional active TB case prevented, compared to targeted screening.

When we assumed that a second, confirmatory positive QFT was needed to initiate LTBI treatment after a positive screen on routine serial testing, overall costs fell slightly for the targeted and annual screening strategies with QFT (Table 6). The number of false positive QFT screens fell dramatically, while true positives in fact increased, because more workers remained eligible for repeat testing (Table 6). However, annual and targeted screening strategies, and the post-exposure QFT strategy, continued to be associated with slightly poorer quality-adjusted survival than the post-exposure TST strategy (Table 6), i.e. the post-exposure TST strategy dominated all others.

**Table 6:** Projected outcomes of six TB screening strategies for a cohort of 1000 healthcare workers: alternate scenario with confirmatory repeat interferon-gamma release assays (IGRAs) (use of two sequential positive IGRAs to identify candidates for treatment of LTBI)

Strategy	Cost in 2015 $CAN	QALYs	New active TB cases	Deaths due to active TB	Deaths due to adverse event to treatment of active TB	Deaths due to adverse event to treatment of LTBI	True positive results	False positive results
Post-exposure screening only								
Tuberculin SKIN Test	$66,387	15,239.98	3.03	0.13	0.0023	0.00036	63	6
QuantiFERON®-TB-Gold	$77,521	15,239.85	2.97	0.13	0.0023	0.00040	67	11
Targeted screening								
Tuberculin Skin Test	$151,517	15,237.96	2.83	0.12	0.0022	0.00093	67	109
QuantiFERON®-TB-Gold	$260,558	15,239.67	2.67	0.12	0.0020	0.00049	78	17
Annual screening								
Tuberculin Skin Test	$404,956	15,231.85	2.68	0.12	0.0020	0.00258	75	413
QuantiFERON®-TB-Gold	$801,059	15,238.94	2.44	0.11	0.0019	0.00072	93	46

*TB* tuberculosis, *QALYS* quality-adjusted life years, *LTBI* latent tuberculosis infection

With the addition of a confirmatory IGRA, the annual TST strategy was dominated by the targeted QFT strategy, with respect to TB case prevention (more expensive, fewer cases prevented). The estimated incremental cost-effectiveness ratio for annual QFT screening compared to targeted QFT screening was $2,350,004 per additional TB case prevented, while for annual QFT screening compared to targeted TST screening it was $1,665,492 per additional TB case prevented.

A threshold analysis indicated that annual screening with the QFT prevents more TB cases than with the TST when the adherence of HCWs with TST reading post-exposure is less than 65.7% (base-case value, 95.0%). The same holds true when the specificity of the TST falls below 95.1% (base-case value, 97.0%), and when the specificity of serial QFT exceeds 96.9% (base-case value, 95.0%). Post-exposure screening only is cheaper with the QFT than with the TST only when the specificity of the TST falls below 83.1% (base-case value, 97.0%). Finally, targeted TST screening is cheaper than post-exposure TST screening only when high-risk HCWs have an annual risk of exposure above 43.6%, which far exceeds any reasonable estimate of exposure in current North American settings.

## Discussion

Our analysis suggests that, in North American settings where patients with TB are likely encountered, routine annual screening for HCWs who perform typical patient care activities provides very limited benefit at high cost, compared to more targeted approaches, provided there is reasonable recognition of exposures after they occur. To prevent less than one additional TB case per 5000 workers over 20 years, the cost of annual screening for intermediate-risk workers is nearly triple that of a targeted strategy where only the highest risk workers are routinely screened. Less intensive screening strategies also appear to be associated with slightly better quality-adjusted survival. These findings reflect the fact that most North American HCWs now face a very small annual risk of TB infection. On a group level, unnecessary LTBI treatment produces small decrements in quality-adjusted survival that outweigh small gains from active TB cases averted in a much smaller number of workers.

In our base case, we assumed that workers were 100% adherent with annual testing, i.e. that it was mandatory for renewal of employment. This conservative assumption may not be applicable to all settings, in which case annual screening would be even less cost-effective compared to the targeted strategies. In addition, steps to ensure adherence with screening after recognised exposures will likely offset any minor decrements in TB prevention if annual screening is stopped. Our results also suggest that the use of QFT is systematically more expensive than the TST, regardless of the screening frequency, and most often does not provide any gain in TB prevention.

In 2015, the World Health Organization published guidelines for screening and management of LTBI in higher-income, lower-incidence countries [32]. The guidelines suggest that HCWs represent one of the groups where testing may be considered; this was a “*conditional*” recommendation, based on “*low to very low quality evidence*” [32]. The guidelines further indicate that screening policy decisions should reflect local epidemiology and context. Along these lines, our findings suggest that, with current epidemiologic conditions in the United States, Canada, and other similar countries, most HCWs derive limited benefit from routine serial screening. On the other hand, annual worker screening will be appropriate, and potentially much more cost-effective, in higher-incidence settings where there is substantial ongoing exposure and infection [33].

With any testing method, false positive results on serial testing [6, 8, 9] lead to a greater number of workers receiving unnecessary and potentially toxic treatment. In addition, they lead to missed opportunities for prevention after significant exposures, potentially contributing to slightly higher numbers of expected cases. Indeed, a recent retrospective cohort study of California first responders estimated a cumulative frequency of positive QFT tests exceeding 25% over 7 years of routine annual testing. The vast majority of these were likely false positives, meaning that individuals who were deemed no longer eligible for subsequent screens could then be missed after true exposure and infection [10]. A recently published Markov modelling analysis, based on extensive review of published data for the QFT test, reinforces these concerns about the possible extent of false positive results with serial testing, and about the potential to miss true positives accordingly [34]. This is distinct from potential advantages of the QFT for single screens.

The use of a second, confirmatory QFT in serial testing leads to fewer false-positive results, and thus fewer individuals placed on unnecessary treatment for LTBI. Our results likewise suggest that it leads to fewer individuals inappropriately excluded from future testing, such that there are ultimately more infections correctly detected among exposed workers. The use of a confirmatory QFT in the annual and targeted screening strategies was associated with higher testing but lower treatment costs, resulting in net savings when compared to treatment after a single positive QFT. There is some possibility that the T-SPOT.TB test may also lead to improved specificity and hence less unnecessary treatment [7], although this was not confirmed in another study [6]. It is also possible that improvements in IGRA manufacturing, and consistent procurement and processing, will lead to better specificity [7].

In recent years, both cohort studies and decision analyses have addressed TB screening of HCWs, but these have focused largely on choice of screening tools, i.e. the use of IGRAs versus the TST [6, 7, 20, 35–38]; they have not directly examined the need for screening as such. As a clinical trial comparing active TB after alternative screening strategies is impossible, a decision analysis is well suited to address the need for screening.

In addition, sensitivity analyses identified key drivers of cost and clinical effectiveness for serial screening. The most important was exposure risk, followed by recognition of exposure, and adherence to post-exposure and annual screening protocols. However, even when we considered high levels of adherence to screening, and high test sensitivity and specificity, cost-effectiveness of annual screening was limited, largely because of low ongoing exposure risk.

del Campo et al. [38] conducted a cross-sectional study of HCWs in Madrid, in a hospital with substantially more admissions for microbiologically confirmed TB (47–66/year) than in most North American hospitals. Participating workers underwent both TST and QFT testing. The authors then used a decision analysis model to project costs and TB cases associated with several potential strategies for testing, based on their study results. They suggested that the most cost-effective strategy for LTBI screening and treatment employed the TST (5 mm cut-off) followed by a confirmatory QFT, with an estimated cost of €14,650 per active TB case prevented (compared to no screening) [38]. However, many differences explain this lower cost estimate. Firstly, the Spanish analysis did not distinguish baseline from subsequent screens, it assumed a high true prevalence of LTBI among workers (30%) and it assumed a constant high risk of progression to active TB among infected workers (2.5%/year). In addition, the strongest predictor of positive test results was employment as a janitor, suggesting that non-occupational factors likely accounted for most of the observed infections, as also suggested by the US registry study of TB in HCWs [12].

As with any decision analysis, our results have several limitations. The most important relates to the quality and variability of published data used for model parameters (probabilities and costs). For this reason, we performed extensive sensitivity analyses, which suggested that our major findings were robust. In addition, we made several simplifying assumptions. For example, while active TB cases were extremely rare among HCWs, we did not model the impact of potential transmission to patients or other workers. This is because, in recent years, the extent and attendant cost of transmission from workers in low-incidence settings has been poorly defined, with a small number of publications detailing extensive contact investigations in extreme circumstances, e.g. a HCW with multi-drug resistant TB. Even in such instances, the true extent of transmission may be difficult to capture [39]. Nonetheless, our analysis suggests that, even if each case of active TB in a worker cost an additional $200,000 for very large-scale contact investigation and treatment, the incremental cost of annual screening would still exceed $1 million per case averted.

We assumed that all cases of active TB in workers would ultimately be diagnosed on the basis of symptoms or other findings. We made this assumption because the goal of screening with the TST or the QFT is to identify and treat latent infection, rather than to diagnose active TB.

We also did not address the cost-effectiveness of baseline screening at hiring, which is needed to identify workers who would benefit from any subsequent tests, and to interpret such tests, regardless of the specific test and frequency adopted. Indeed, the continued use of baseline tests is also essential in identifying the much larger group of HCWs who begin employment with latent TB infection, and who may be suitable candidates for treatment accordingly. Most active TB in US HCWs now appears to reflect infection acquired outside the workplace (e.g. before hire). Foreign-born HCWs have a ten-fold higher TB incidence than US-born HCWs, and both groups now have TB incidence rates similar to their non-HCW counterparts in the general population [12]. Baseline testing is even more important in areas where a large proportion of workers are foreign-born, such as Western European countries. Extended residence and/or previous healthcare work in countries with higher TB prevalence are also highly relevant to baseline TB risk. The use of newer LTBI treatment regimens, such as isoniazid-rifapentine [40] or rifampin [41], will enhance the impact of baseline testing, to the extent that treatment adherence improves and serious adverse events become less frequent.

US data suggest that only about 14.5% of US HCWs are foreign-born [12], while in an older nationwide Canadian study, 18% of workers had positive TSTs at hiring [13]. More recent data from US cohorts report 95% or more to have negative baseline tests [6, 7, 11]. This implies that most North American HCWs are expected to have negative baseline tests, and hence, in many settings, the number potentially eligible for repeat annual testing is large. Reducing the number of workers retested could provide substantial savings to occupational health and TB prevention programmes.

## Conclusions

For most North American HCWs, annual screening for latent TB infection appears expensive with limited health gains. IGRAs improve the specificity of baseline testing, but are unlikely to improve the cost-effectiveness of subsequent screens due to low annual risks of infection. Annual worker screening may no longer be appropriate in most settings, and reconsideration of this longstanding recommendation may be warranted. The resources currently allocated to routine TB testing for HCWs may be more productively used for other TB prevention activities.
